# Altered Functional Connectivity and Complexity in Major Depressive Disorder after Musical Stimulation

**DOI:** 10.3390/brainsci12121680

**Published:** 2022-12-07

**Authors:** Pintao Qiu, Jinxiao Dai, Ting Wang, Hangcheng Li, Cunbin Ma, Xugang Xi

**Affiliations:** 1 HDU-ITMO Joint Institute, Hangzhou Dianzi University, Hangzhou 310018, China; 2 Key Laboratory of Brain Machine Collaborative Intelligence of Zhejiang Province, Hangzhou 310018, China; 3 School of Automation, Hangzhou Dianzi University, Hangzhou 310018, China; 4 Hangzhou Mingzhou Naokang Rehabilitation Hospital, Hangzhou 311215, China

**Keywords:** major depressive disorder, music therapy, electroencephalography, functional connectivity, phase-locking value, frequency band, brain network

## Abstract

Major depressive disorder (MDD) is a common mental illness. This study used electroencephalography (EEG) to explore the effects of music therapy on brain networks in MDD patients and to elucidate changes in functional brain connectivity in subjects before and after musical stimulation. EEG signals were collected from eight MDD patients and eight healthy controls. The phase locking value was adopted to calculate the EEG correlation of different channels in different frequency bands. Correlation matrices and network topologies were studied to analyze changes in functional connectivity between brain regions. The results of the experimental analysis found that the connectivity of the delta and beta bands decreased, while the connectivity of the alpha band increased. Regarding the characteristics of the EEG functional network, the average clustering coefficient, characteristic path length and degree of each node in the delta band decreased significantly after musical stimulation, while the characteristic path length in the beta band increased significantly. Characterized by the average clustering coefficient and characteristic path length, the classification of depression and healthy controls reached 93.75% using a support vector machine.

## 1. Introduction

Major depressive disorder (MDD) is a common multidimensional disorder [1,2]. Symptoms often include lack of energy and loss of interest in life [3]. Compared with other diseases, MDD mainly impairs social cognitive function [4,5,6]. As a major worldwide disease, the treatment of MDD is usually divided into drug therapy and psychotherapy [7,8]. Although drugs are the main treatment for depression, 20% of patients cannot take them because of strong adverse drug reactions or psychological problems [9]. Psychotherapy as an alternative option, such as through music therapy, has been shown to significantly relieve depression in clinical studies [10]. Numerous studies have shown that the expression of music can improve the functioning of the nervous and endocrine systems [11,12]. At the same time, music therapy also provides patients with the opportunity to express their inner emotions through music, which can enable patients to obtain opportunities for self-expression through musical activities [13,14]. Therefore, musical stimulation therapy has been proven to be a new approach in the treatment of MDD patients, and the effect is widely recognized [15,16,17].

Music therapy is a great help in treating MDD [18,19,20], but the specific changes in brain connectivity that this treatment modifies are unknown. Electroencephalography (EEG) is a non-invasive real-time neuroimaging technique. Benefiting from its high temporal resolution, EEG records electrical activity in relation to neuronal activity with excellent temporal resolution on the order of milliseconds. These advantages of EEG make it an irreplaceable technique when observing the brain [21]. EEG, combined with graph-theory-based network analysis, is gradually being used to explain the topology of brain networks and to analyze functional connections in the brain [22]. Functional connectivity (FC) is defined as the statistical interdependence between spatially distant neurophysiological regions, most commonly the correlation or coherence assessment between signals from pairs of electrodes [23]. FC provides a new approach to the rehabilitation of MDD patients [24]. Most of the research on MDD is based on non-invasive imaging, such as fMRI and EEG [25,26]. Signal features, including FC, are extracted by these techniques to understand data on normal or pathological brain states. EEG research focuses on MDD feature extraction and classification [27,28,29]. EEG-based signal feature extraction and classification of MDD patients focuses on the resting state or repetitive task state, which is different from the test state under natural conditions, as the world in which people live is changeable. As a part of daily entertainment, music can improve people’s spiritual world, open closed hearts, and relieve depression [30,31]. Music therapy has emerged as a powerful tool in the treatment of MDD, and previous research has focused on exploring differences in how the brain responds to musical stimuli [32,33]. However, these approaches have yet to explain what the music changes to dampen the brain’s hypoactive state. By combining EEG with musical stimulation therapy and decomposing EEG signals into different frequency bands, differences in brain function in response to musical stimulation were revealed [34,35,36,37]. When people are in different active states, there are differences in EEG signals of different frequencies [38,39]. Previous studies have demonstrated that the FC of MDD changes in different frequency bands, so the diagnosis of MDD by FC analysis with different frequency bands is worth investigating [40]. Specific frequency changes and specific network connectivity changes were seen in behavioral cognitive tasks, especially during music perception. According to He et al., music therapy relieves clinical MDD symptoms by improving functional brain connectivity and increasing connectivity with changes in FC [41,42]. These results support our hypothesis that functional connectivity across frequency bands is altered during musical stimulation therapy in MDD patients. Nonetheless, the mechanisms underlying functional connectivity problems and brain responses during musical stimulation therapy in MDD patients are unclear.

In the EEG sensor space, the brain network consists of nodes and edges, where electrodes represent nodes and channel connections represent edges [43,44]. Various network properties are valid measures for quantifying functional integration and functional segregation in the brain. At present, the feature matrix of EEG functional connectivity mainly adopts correlation and coherent coupling methods. Phase-locking value (PLV) and coherence have stable effects on bulk conductivity [45]. Therefore, this paper adopts the PLV algorithm to calculate the correlation between EEG channels. By comparing the results of functional linkages, biomarkers that distinguish MDD and control groups were extracted.

This study mainly analyzes the network changes of MDD in different frequency bands before and after musical stimulation. The connectivity matrix and topology graph are compared, and the network properties of graph theory are analyzed. The network features mainly include path length, node degree and clustering coefficient.

## 2. Materials and Methods

### 2.1. Participants

A total of 16 subjects were selected for this study, including 8 patients with major depressive disorder and 8 healthy controls. All subjects signed written consent. The ethics review was approved by the Ethics Committee of Hangzhou Mingzhou Naokang Rehabilitation Hospital, approval number 20210201. Of the 8 patients selected, 6 were male and 2 were female. Age: 24–51 years (mean ± SD: 30.85 ± 7.5 years). Patients with severe depression were from the Seventh People’s Hospital of Hangzhou. The patients were surveyed with a depression scale. Questionnaires included the Generalized Anxiety Disorder (GAD-7) and Patient Health Questionnaires (PHQ-9). The short international interview exam for neuropsychiatric disorders was used to screen patients for depression. The main criteria were the Diagnostic and Statistical Manual of Mental Disorders-IV (Dsm-5) fourth edition criteria. The inclusion criteria for major depressive disorder were: patients with PHQ-9 scores greater than 10 and GAD-7 scores greater than 10. All participants signed an informed consent form. To exclude cognitive impairment, the educational background of subjects was required to be reported. The education level of the patients was divided into two levels: 2 with a master’s degree and 6 with a bachelor’s degree.

Eight healthy controls (HC) were selected for the experiment, aged from 24 to 48 years old (mean ± SD: 27.65 ± 8.6 years old), and all were male. They all had college degrees. Demographic variables of depressed patients and healthy controls are shown in Table 1. The inclusion criteria for healthy control samples included:

(1) Age matching with depression patients.
(2) In good physical and mental health, with no history of mental illness.
(3) A PHQ-9 questionnaire score of less than 4.
(4) Exclusion of individuals with chronic diseases.
(5) Cognitively normal, no history of major depression, schizophrenia, bipolar disorder, or drug abuse, and no medication that may affect cognition and walking.

### 2.2. EEG Data Acquisition and Preprocessing

64-channel (NeuSen.W64, Neuracle, Changzhou City, China) EEG signals were collected at a sampling frequency of 500 Hz. The instrument used in this study adopted the 10–20 international EEG standard. Before the electrodes were connected, each subject cleaned the area of contact. During the experiment, the impedance of each electrode was maintained below 15 KΩ. In order to visualize the whole-brain functional connectivity activity, the Fp1, Fp2, Fpz, F3, F4, F7, F8, Cz, C3, C4, T7, T8, Pz, P3, P4, P7, P8, O1 and O2 channels were retained, and the remaining channels were excluded.

We used the EEGLAB toolbox for EEG data processing. EEGLAB is an open-source MATLAB toolkit that uses a visual interface to rapidly process biological signals [46]. The EEG signal whose re-reference form was a common average reference was preprocessed using the EEGLAB toolbox. All EEG signals were bandpass filtered using finite impulse response filters with cutoff frequencies of 0.1 Hz and 30 Hz. Any 50 Hz powerline interference was eliminated using EEGLAB’s independent components. Additionally, abnormal fragments were removed manually. Similarly, blinking and eye movements were eliminated manually by visual examination as well as by using independent component analysis techniques. Here, components that represented artifacts, such as blinking, eye movements and muscle activity, were removed. Typically, for each subject, only one or two independent components associated with blinking or eye movements were removed, and the remaining components were used for analysis.

### 2.3. Experimental Paradigm

**Figure 1:**
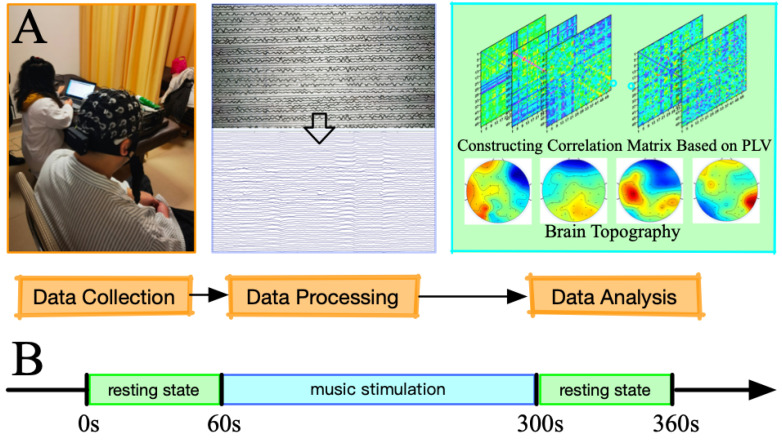
EEG acquisition process. (**A**) Experimental flowchart; (**B**) time distribution of musical stimulation.

In this experiment, the EEG data of healthy controls and depressed patients were collected. EEG signals were collected while the subjects were in resting state. The experiment was divided into three parts, totaling six minutes. The specific steps are as follows: (1) the patient’s resting state data for 1 min in the relaxed state was collected; (2) music audio was played to stimulate the patient for 4 min; (3) 1 min resting EEG signal was collected again after musical stimulation. The EEG acquisition process is shown in Figure 1. The music signal used in the experiment was a piece of soft music selected by hospital experts conducive to mental relaxation. Music standards were recommended by doctors and voted on by all participants. The final choice was Canon in D Major, author Dylanf.

### 2.4. Data Analysis

#### 2.4.1. Phase Locking Value

Phase-locking value (PLV) statistics are a time course, so phase-locking values can be used to observe instantaneous changes in connectivity [47]. The synchronization algorithm, based on Hilbert transform, has been widely used in correlation analysis between multi-channel EEG signals. In this experiment, the phase was used to calculate the PLV, and the Hilbert transform $\tilde{x}\left(t\right)$ of the signal $x\left(t\right)$ is defined as:

(1) $$\tilde{x}\left(t\right)=H\left[x\left(t\right)\right]=\frac{1}{\pi }\overset{+\infty }{\underset{-\infty }{\int }}\frac{x\left(\tau \right)}{t-\tau }d\tau =x\left(t\right)*h\left(t\right)$$

For a single-channel EEG signal *x*(*t*), the analytical signal can be defined as:

(2) $$\begin{array}{c} Z\left(t\right)=x\left(t\right)+i\tilde{x}\left(t\right)=A_{x}^{H}\left(t\right)\exp\left[i\Phi_{x}^{H}\left(t\right)\right] \end{array}$$

where $\Phi_{x}^{H}$ is the phase of $x\left(t\right)$ and $A_{x}^{H}$ is the magnitude of $x\left(t\right)$. $\exp\left[i\Phi_{x}^{H}\left(t\right)\right]$ is the complex signal obtained with the help of phase by Euler’s formula. The PLV of this study was defined as:

(3) $$\begin{array}{c} PLV=\sqrt{{<cos_{\Phi_{xy}^{H}\left(t\right)}}_{t}^{2}>+{<sin_{\Phi_{xy}^{H}\left(t\right)}}_{t}^{2}>} \end{array}$$

where $\Phi_{xy}\left(t\right)=\phi_{x}\left(t\right)-\phi_{y}\left(t\right)$ represents the phase difference of the two signals. If PLV = 0, $x\left(t\right)$ and $y\left(t\right)$ are not synchronized, and if PLV = 1, it means that the two signals are completely synchronized. $<{>}_{t}$ represents the average over time.

Determining the adjacency matrix based on the PLV is defined as:

(4) $$\begin{array}{c} w_{pq}^{*}=\left\{\begin{array}{c} \frac{1}{s}\left|\sum_{n=1}^{s}exp(i\left(\Delta \Phi_{n}\left(t\right)\right)\left.)\right|,if\;x\neq y\right.\\ 1\;\;\;\;\;\;\;\;\;\;\;\;\;\;\;\;\;\;\;\;\;\;\;\;\;\;\;\;\;\;\;\;\;\;\;\;\;\;\;\;\;\;\;\;\;\;\;,if\;x=y \end{array}\right. \end{array}$$

where S is the length of the time series.

#### 2.4.2. Network Characteristics

The constructed PLV-based functional brain network was used to characterize the connection structure between the MDD brain channel pairs under different musical stimuli. The building process of the brain functional network can be thought of as a process of abstractly representing connections between brain regions as nodes and connecting edges. The analysis of brain functional segregation is based on the local scale, and local measures focus on the functional differentiation capacity of the brain and measure the clustering properties within the brain network [48]. Because the brain functional network is complex and changeable, in order to further monitor the functional state of the brain, graph theory is used to extract multiple topological properties of the brain functional network: the brain functional network is constructed to extract the clustering coefficients and characteristic path lengths under different bands [49,50,51]. In this study, the corresponding local features are extracted for each PLV network constructed.

The degree in the network represents the number of connections between a node and other nodes, and can be used as a measure to analyze the importance of a node in the network. The clustering coefficient of the brain function network indicates the possibility that the neighbors of a node become neighbors of each other [52,53,54]. The clustering coefficient (CC) of a node can reflect the closeness of the association relationship between the node groups in the network and is defined as:

(5) $$\begin{array}{c} CC=\frac{1}{n}\sum_{i\in N}\frac{2t_{i}}{k_{i}\left(k_{i}-1\right)} \end{array}$$

where n is the total number of nodes, $k_{i}$ represents the number of adjacent nodes that the node has, and $t_{i}$ represents the number of relationships between the network through i-neighbor nodes.

The characteristic path length is the average of the shortest path lengths between two nodes [55]. The length of the characteristic path can measure the information interaction strength between two nodes. The shorter the length, the stronger the information interaction between the two nodes, which can effectively characterize the connectivity of the two nodes. The characteristic path length (CPL) is used to measure the robustness of the network topology, and it is defined as:

(6) $$\begin{array}{c} CPL=\frac{1}{n}\sum_{i\in N}\frac{\sum_{j\in N,j\neq i}d_{ij}}{n-1} \end{array}$$

where n represents the number of nodes in the network, i and j represent different types of nodes in the network, and $d_{ij}$ represents the distance between point i and point j.

## 3. Results

### 3.1. Network Analysis

**Figure 2:**
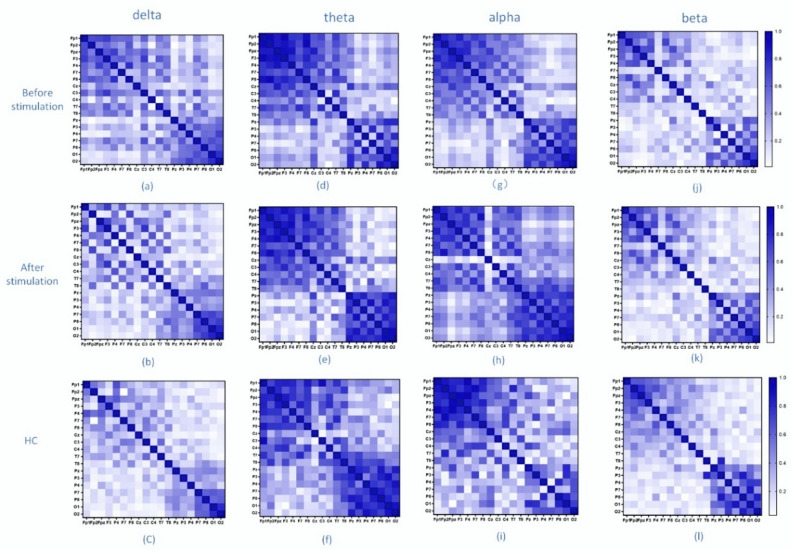
Before and after musical stimulation, depressed patients and healthy controls had different frequency band association matrices. Each row shows the state of the subject, and each column shows the frequency band of the signal (**a**–**l**).

Figure 2 shows the correlation matrix constructed using the PLV of the EEG signals for MDD (before and after music stimuli) and HC in four frequency bands: delta (0.5–4 Hz), theta (4–8 Hz), alpha (8–13 Hz), and beta (13–30 Hz). In the delta band, the phase lag index of depressed patients was higher than that of healthy controls, and the phase lag index of depressed patients decreased in multiple channels after the musical stimulation. In the theta band, the phase lag index was slightly higher in healthy controls than in depressed patients in the parieto-occipital lobe. However, after musical stimulation, the phase lag index of depressed patients showed different changes in different regions, although there was an overall increase, but no obvious regularity. In the alpha band, the phase lag index of the EEG signals in depressed patients increased significantly in the parietal and occipital regions and slightly in the frontal and polar regions after musical stimulation, but decreased in other locations. Compared with the healthy control group, the phase lag index of depressed patients in the alpha frequency band was decreased overall, and some regions were higher than the control group. In the beta band, the index of the healthy control group was higher than that of depressed patients, and after musical stimulation, the phase lag index of depressed patients also increased. According to the matrix, it can be found that in the beta band, the phase lag index changed significantly with significant regularity.

**Figure 3:**
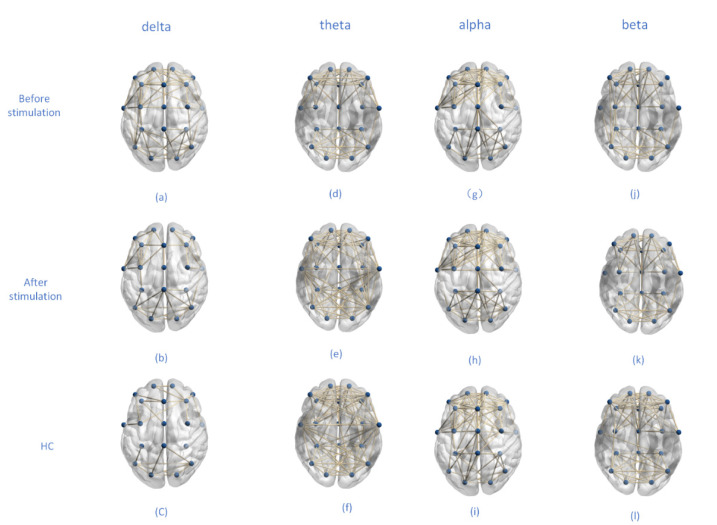
Brain network topology of depressed patients and healthy controls at different frequency bands before and after musical stimulation. Each row shows the state of the subject, and each column shows the frequency band of the signal (**a**–**l**). The blue dots represent the poles of light, and the yellow lines represent the connections of the channels.

In order to show the connection of brain regions more clearly, we drew a network topology map using the binary sentence method as shown in Figure 3. In the delta band, the association of depressed patients decreased after musical stimulation, mainly in the frontal pole and frontal lobe, and also in the parietal lobe. Healthy controls were less connected to this band than depressed patients. In the theta band, the topological connection increased after musical stimulation, and the connection in the central region increased. In the alpha band, depressed patients showed improved left–right brain symmetry and slightly improved connectivity between the frontal and central regions after musical stimulation. Healthy controls and musical stimulation did not change much in the brain topology of depression. In the beta band, after musical stimulation, the functional topology connectivity of depressed patients’ brains was reduced, mainly in the frontal lobe and central region, especially in the left hemisphere. Healthy controls were also less connected than depressed patients. In this study, the threshold was 80% of the maximum value.

### 3.2. Network Properties

**Figure 4:**
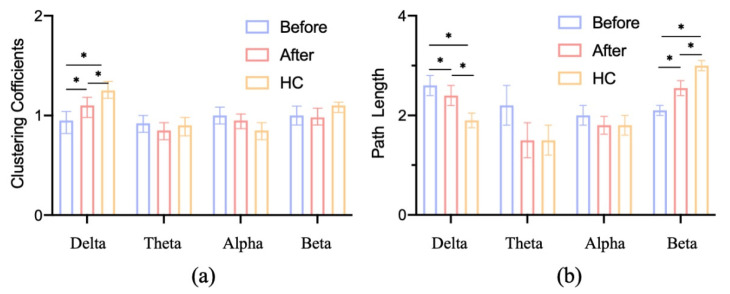
EEG functional brain network results. (**a**) Clustering coefficient, (**b**) characteristic path length. * *p* < 0.05, data are expressed as mean ± SEM.

The average clustering coefficients of depressed patients before and after musical stimulation and healthy controls in different frequency bands are shown in Figure 4a. As can be seen from the Figure, in the delta band, the clustering coefficient increased significantly after musical stimulation, and the clustering coefficient was higher in depressed patients than in healthy controls (with a significant difference). In other frequency bands, the average clustering coefficient of depressed patients decreased after musical stimulation, but the difference was not significant.

The characteristic path length of the brain functional network in depressed patients before and after musical stimulation, and healthy controls at each frequency band, is shown in Figure 4b. In the beta band, the characteristic path length of the brain functional network in depressed patients increased significantly after musical stimulation, while the characteristic path length of other bands decreased. In addition, compared with healthy controls, the characteristic path length of depressed patients in the beta band was significantly lower than that of healthy samples, while the characteristic path length in the delta band was significantly higher.

**Figure 5:**
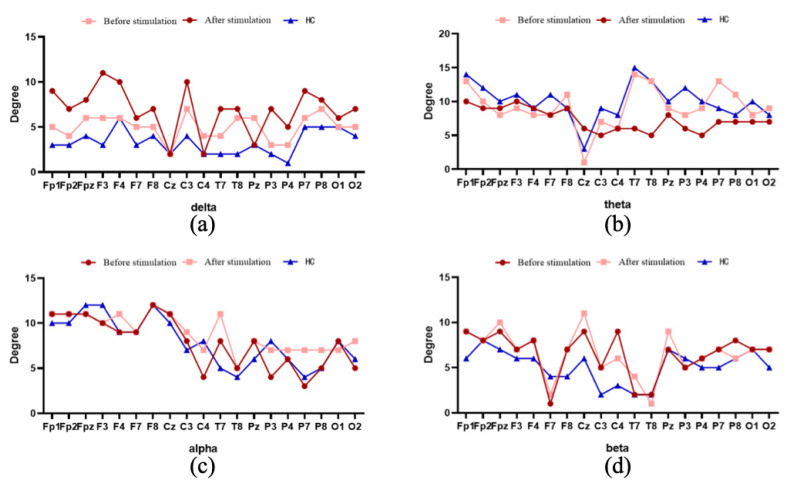
Degree of nodes in EEG functional brain network. (**a**) Delta band; (**b**) theta band; (**c**) alpha band; (**d**) beta band. The horizontal axis represents the channel position, and the vertical axis represents the number of nodes.

The number of node degrees in different frequency bands in the brain functional network of depressed patients and healthy controls is shown in Figure 5. In the delta band, the values of all node degrees decreased in the depressed patients after musical stimulation, and the node degrees were much lower in the normal control group than in the depressed patients. In the theta band, the node degree in the temporal lobe region (T7, T8) was significantly increased in depressed patients compared with healthy controls. In the alpha frequency band, there was no obvious difference in node degree of the brain functional network among different states. In the beta band, the node degree of the central region (Cz, C3, C4) was significantly different between depressed patients and healthy controls, and the musical stimulation had little effect.

### 3.3. Classification

The main purpose of this section is to classify depression patients and the healthy control group, and discuss the effect of feature recognition from individual directions. According to the above analysis, the average clustering coefficient of the delta band and the characteristic path length and the characteristic path length of the beta band were adopted as the effective features of recognition. Features were classified using Support Vector Machine (SVM), Decision Tree (DT), K-Nearest Neighbor (KNN) and Random Forest (RF) algorithms [56,57,58,59,60]. Ten-fold cross-validation was used. The classification results are shown in Table 2:

## 4. Discussion

This study explores the effect of musical stimulation therapy on functional brain connectivity in patients with MDD. Specifically, first, subjects’ EEGs before and after music therapy were recorded and preprocessed. Secondly, a PLV-based correlation matrix was constructed according to frequency segmentation, and the phase lag index was used as an evaluation criterion for analysis. In order to intuitively characterize the connectivity of brain regions, we drew the network topology corresponding to the binary matrix. Then, the characteristic path length and average clustering coefficient of the brain function network in each frequency band of depression patients and the healthy control group before and after musical stimulation were used as indicators to calculate the network characteristics. Finally, four machine learning methods were used to classify patients with depression and healthy controls, and the effect of feature recognition was discussed from an individual perspective.

According to Figure 2 and Figure 3, we can analyze the changes in the brain networks before and after musical stimulation. In the delta band, the associated connections in depressed patients were reduced after musical stimulation, and were mainly concentrated in the frontal pole and frontal lobe regions. Studies have shown that delta signals brain abnormalities to a certain extent. The frontal lobe of the brain is responsible for higher levels of thought, including language, emotional expression, and working memory. This partly explains the lower connectivity of the frontal lobe region in the delta band in healthy patients than in depressed patients. The functional network in the delta band may be an important band to distinguish between types of depression. In the theta band, the change in the topology was not obvious. Changes in the alpha band can be analyzed using alpha asymmetry. Alpha is a frequency band that indicates brain health and is strongly associated with depression, and alpha asymmetry is also a biological feature of depression [61,62]. In the beta band, after musical stimulation, the functional topology connectivity of depressed patients was reduced, mainly in the frontal lobe and central region. The reason for this phenomenon may be that beta bands indicate the degree of emotional change and reflect the level of arousal in the brain. In addition, the cluster degree of the healthy control group also decreased in this frequency band, and the difference between the depressed patients and the healthy control group was significant.

The frequency is related to the frequency of the musical stimulus [40]. According to the analysis of the statistical results, for the clustering coefficient, there was a significant difference in the delta frequency band before and after the musical stimulation, and there was also a significant difference between the patients and the control group. For the characteristic path length, there was a significant difference between the delta and beta bands. The results obtained were similar to previous studies [28].

Figure 4 and Figure 5 show the changes to network properties. For average clustering coefficient, the clustering coefficient, which measures the degree of aggregation of nodes in the functional brain network, meant that depressed people were less connected in the delta band and improved after musical stimulation. In other bands, there was little difference between healthy controls and depressed patients. Based on the above analysis, the clustering coefficient in the delta band can be used as a feature to classify depression and health samples. According to Figure 4, since the path length shows the path from a node to another node, the shorter path length also represents the higher information transmission efficiency, indicating that in the beta band, depressed patients have a poor information transmission rate and a tendency of random network. In the theta and alpha bands, the difference between healthy samples and depressed patients was small, making it difficult to analyze their information transmission efficiency.

According to the classification results, SVM had the best classification effect, reaching 93.75%, followed by KNN at 81.25%, and DT had the worst classification performance of only 68.75%. In addition, SVM also had the highest accuracy and recall rate among the classifiers, which shows that SVM has unique advantages in solving the problem of small samples and nonlinear dichotomy. Therefore, the clustering coefficient and characteristic path length can be considered as biomarkers to distinguish MDD from healthy controls.

This study has some limitations. First of all, EEG signals are very susceptible to ex-ternal interference. Although we collected signals in a quiet environment and supplemented the preprocessing, there were still some interferences that we could not completely eliminate. Second, the number of subjects were relatively small, and the decision tree appears to be over-under-fit. Therefore, we need more samples to further verify the results. Finally, the choice of depression assessment scale was too singular, and we can consider choosing MADRS or HAM-A, which is actively implemented by doctors and can analyze individual items. Music therapy has been shown to be effective in the treatment of depression, and future research can consider the effects of different styles of music on depression.

## 5. Conclusions

This study obtained the following three results. First, delta and beta band connectivity decreased after musical stimulation, while alpha band connectivity increased. Secondly, in terms of EEG functional network characteristics, the average clustering coefficient, characteristic path length and degree of each node in the delta band decreased significantly after musical stimulation, while the characteristic path length in the beta band increased significantly. Finally, the average clustering coefficient and characteristic path length can be used as features to distinguish MDD from healthy controls.

## Figures and Tables

**Table 1:** Demographic variables of depression patients and healthy controls.

	MDD (n = 8)		HC (n = 8)		*p*
	Average	SD	Average	SD	
age	30.85	7.5	27.65	8.6	0.89
gender	6 male/2 female		8 male		
PHQ-9	15.42	5.32	2.44	0.92	0.00
GAD-7	11.62	6.50	2.19	3.74	0.00

**Table 2:** Classification accuracy of association matrix.

Classifier	Accuracy	Precision	Recall
KNN	81.25%	75%	68.75%
SVM	93.75%	87.5%	93.75%
DT	68.75%	62.5%	62.5%
RF	75%	68.75%	75%

## Data Availability

The data presented in this study are available on request from the corresponding author. The data are not publicly available due to privacy or ethical restrictions.
